# Recent Advances in the Chemobiological Upcycling of Polyethylene Terephthalate (PET) into Value-Added Chemicals

**DOI:** 10.4014/jmb.2208.08048

**Published:** 2022-10-13

**Authors:** Joyce Mudondo, Hoe-Suk Lee, Yunhee Jeong, Tae Hee Kim, Seungmi Kim, Bong Hyun Sung, See-Hyoung Park, Kyungmoon Park, Hyun Gil Cha, Young Joo Yeon, Hee Taek Kim

**Affiliations:** 1 Department of Food Science and Technology, Chungnam National University, Daejeon 34134, Republic of Korea; 2 Department of Biochemical Engineering Gangneung-Wonju National University, Gangneung 25457, Republic of Korea; 3 Synthetic Biology Research Center, Korea Research Institute of Bioscience and Biotechnology, Daejeon 34141, Republic of Korea; 4 Department of Biological and Chemical Engineering, Hongik University, Sejong 30016, Republic of Korea; 5 Center for Bio-based Chemistry, Korea Research Institute of Chemical Technology (KRICT), Ulsan 44429, Republic of Korea

**Keywords:** Polyethylene terephthalate (PET), substrate production for bioconversion, biological upcycling, value-added chemicals

## Abstract

Polyethylene terephthalate (PET) is a plastic material commonly applied to beverage packaging used in everyday life. Owing to PET’s versatility and ease of use, its consumption has continuously increased, resulting in considerable waste generation. Several physical and chemical recycling processes have been developed to address this problem. Recently, biological upcycling is being actively studied and has come to be regarded as a powerful technology for overcoming the economic issues associated with conventional recycling methods. For upcycling, PET should be degraded into small molecules, such as terephthalic acid and ethylene glycol, which are utilized as substrates for bioconversion, through various degradation processes, including gasification, pyrolysis, and chemical/biological depolymerization. Furthermore, biological upcycling methods have been applied to biosynthesize value-added chemicals, such as adipic acid, muconic acid, catechol, vanillin, and glycolic acid. In this review, we introduce and discuss various degradation methods that yield substrates for bioconversion and biological upcycling processes to produce value-added biochemicals. These technologies encourage a circular economy, which reduces the amount of waste released into the environment.

## Introduction

Plastics are ubiquitous in daily life as they are used in packaging, construction, and clothing [1]. These materials are significant in modern society owing to their adaptability, low manufacturing costs, and desirable physical features, which include flexibility, lightness, impermeability, and durability [2, 3]. Consequently, the demand for plastics has continuously increased; however, improper recycling of plastic waste causes plastic pollution [4]. Plastic accumulation threatens human survival and the environment [5]. According to Geyer *et al*. [6], as of 2015, 6,300 million metric tons of plastic waste have been disposed of worldwide, and this amount is increasing by 3%annually. Of all the entire plastic waste generated, only 9% is recycled, 79% is landfilled or discarded into oceans, and 12% is incinerated [7]. Owing to the low rate of recycling and natural disintegration, plastic usage has caused various environmental issues [8], such as microplastic creation in marine and terrestrial ecologies and plastic island evolution [9]. Approximately 5-13 million tons of plastic are estimated to end up in the ocean each year, and 5 trillion plastic particles, which are fatal to aquatic life, now float in the world’s oceans [2, 10]. Plastic waste is primarily downcycled into less recyclable and cheap products like garden furniture and plant pots [7]. Currently, with the changing living conditions attributed to the COVID-19 pandemic, plastic production and pollution have been on the increase, and with the low recycling rate, the effects of this pollution continue to worsen and further endanger the environment. Therefore, such man-made disasters must be rapidly mitigated.

Reuse, incineration, and landfilling are conventional methods for managing plastic waste [9]. Plastic incineration provides energy but also emits particulate matter and toxic gases such as unburned hydrocarbons, nitrous and sulfurous oxides, furans, and dioxins, which cause severe environmental damage [11] For these reasons, landfilling and incineration are not recommended for plastic garbage disposal, and instead, more sustainable and environmentally friendly methods are required [12].

Due to its exceptional mechanical properties, thermal stability, and impermeability to gases and liquids, polyethylene terephthalate (PET) is the most popular synthetic plastic material applied in beverage packaging, food containers, bottles, and textiles [13]. PET is produced by polycondensation of terephthalic acid (TPA) and ethylene glycol (EG) or trans-esterification of dimethyl terephthalate (DMT) and EG. Its stability and resistance to hydrolytic or enzymatic degradation have led to PET being the most commonly found plastic waste in the environment [1]. Currently, PET is primarily recycled via physical and chemical methods; various studies have also reported the use of biodegradation using microorganisms to break down PET into its monomers: TPA and EG. Physical recycling involves heat treatment at high temperatures, and PET is degraded into downcycled products because of the loss of mechanical properties, which are different from those of virgin PET [1]. Chemical recycling uses chemicals and expensive catalysts to decompose waste into monomers; recycled plastics are typically more expensive than virgin plastics and are not economically viable [13]. To solve the plastic pollution problem, various approaches, including biological depolymerization and upcycling of plastic wastes, and even plastic-eating microbes, have been considered [14]. Circular repurposing of plastic materials is critical for building a socioeconomic ecosystem without contributing to plastic waste [15]. Upcycling converts waste materials into products of greater worth and quality in their second life [16]. Upcycling also helps achieve the circular economic feasibility of plastic waste with complete recyclability and no loss of value or usability, in contrast to other recycling methods.

**Fig. 1:**
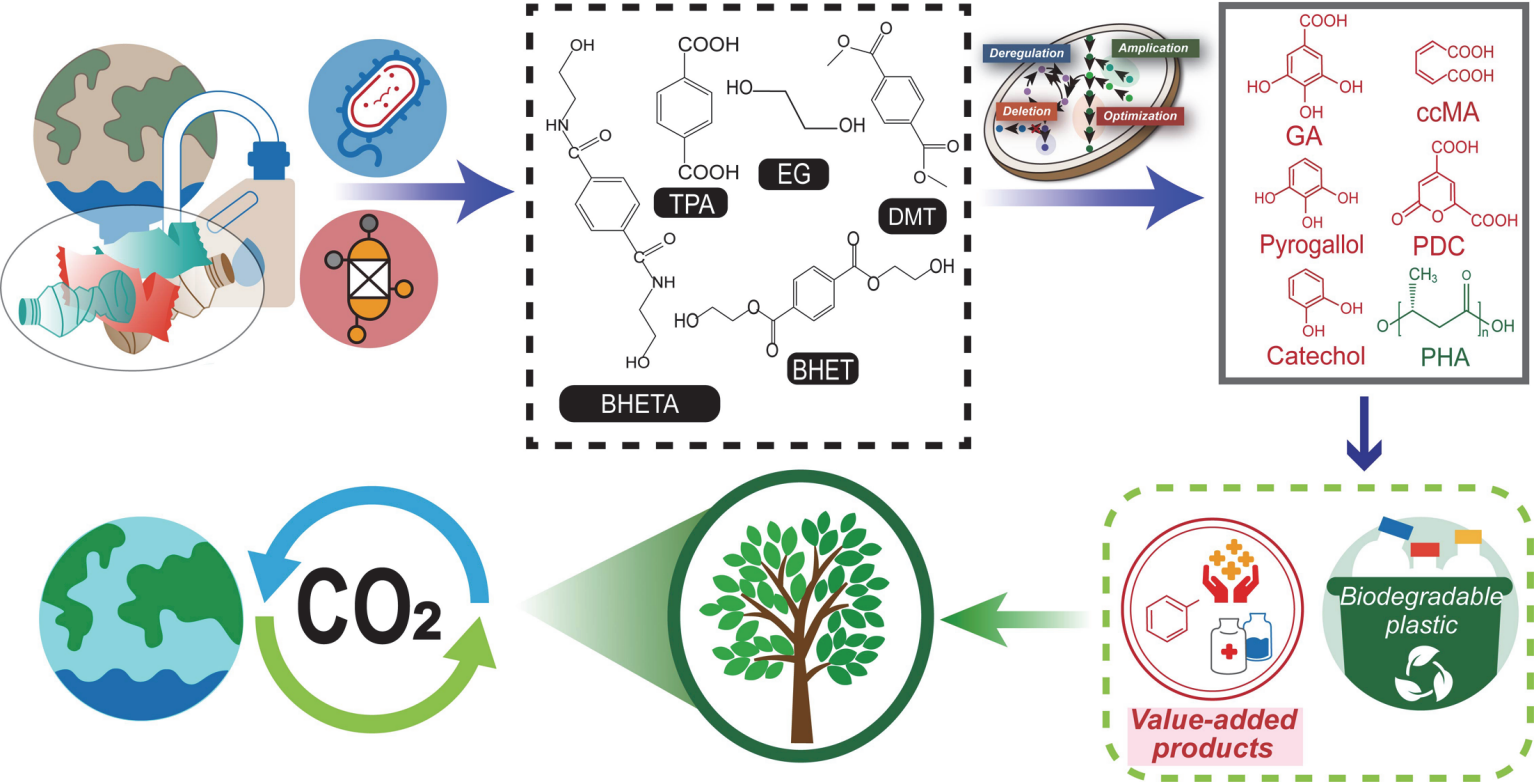
Overall scheme representing the primary aspects of the review.

In this review, we discuss the development of the upcycling processes suitable for PET, comprising two steps: 1) substrate production from PET waste, and 2) upcycling of the substrate into value-added chemicals (Fig. 1). For producing substrates by degrading PET, we suggest processes suitable for bioconversion to obtain value-added chemicals after reviewing various existing processes, including pyrolysis, gasification, and depolymerization, along with their advantages and disadvantages. Following the review of chemical and biological upcycling models, we propose an efficient upcycling model for producing value-added chemicals. Finally, we suggest a method for PET upcycling to achieve economic feasibility and a circular economy regarding the plastic life cycle, as a means to address plastic environmental pollution.

## Production of Substrates from PET Waste for Bioconversion Processes

**Fig. 2:**
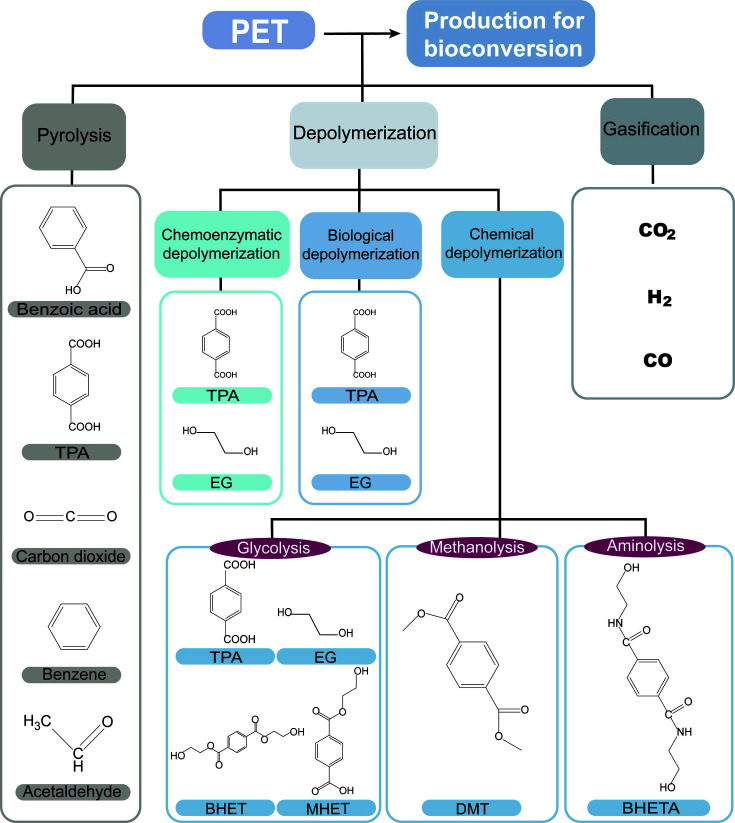
Substrate production processes from PET waste into respective products, including different processes used to produce substrates that are employed in biological upcycling.

Various processes, including pyrolysis, gasification, and depolymerization, can be applied to produce substrates from PET waste (Fig. 2, Table 1). Although individual processes for substrate production from PET waste are well established, selecting a suitable upcycling process, particularly regarding biological upcycling, is crucial because of the economic feasibility and establishment of refineries. Therefore, in this section, we discuss the status of research, along with the advantages and disadvantages, to suggest a process suitable for upcycling.

## Substrate Production by Pyrolysis of PET

Pyrolysis is the thermal transformation of polymers into liquid fuels and value-added products at considerably high temperatures (300-900°C) in the absence of oxygen [17-19]. PET pyrolysis releases products such as TPA, vinyl terephthalate, aromatic compounds (namely toluene and benzene); esters such as vinyl benzoate; carboxylic acids such as ethyl and methyl benzoic acid; and aliphatic hydrocarbons such as ethane and methanol. TPA formed during pyrolysis can be used for upcycling after purification; however, TPA clogs the equipment [20]. Pyrolysis is affected by factors like substrate type and time, and temperature, which influence the polymer degradation rate; and catalyst loading, which improves the process efficiency through time reduction. Slow, fast, and flash pyrolysis methods are based on reaction times, and microwave pyrolysis is based on the apparatus type [21].

PET waste was pyrolyzed for energy recovery at 500°C, yielding solids, gas, and residue products of 24.33, 35.67, and 74.67 wt%, respectively [22]. The effect of plastic waste loading was studied using microwave co-pyrolysis of PET and rice husk mixtures at 600°C for 4-13 min, which yielded syngas (29%) affected by increasing plastic waste loading, biocrude, and char [23]. Slow pyrolysis of PET waste was conducted at 400°C producing gas, solid residue, and oil at 13.3 ± 0.6, 39.7 ± 1.6, and 46.7 ± 1.9 wt%, respectively [24]. Catalytic pyrolysis of PET was performed to study the effect of temperature and catalyst on PET degradation (500°C in a fixed-bed, semi-batch reactor using a Lewis-Brønsted acid side catalyst). The increase in temperature increased the PET conversion rate, forming liquid-, gas-, and solid-phase products with benzoic acid and TPA. This process is primarily affected by the formation of char, which influences the conversion rate and product yield [25]. Pyrolysis does not require intense waste sorting and feedstock pre-treatment, and is consequently convenient and flexible; hence, it is economical and less labor-intensive. Moreover, pyrolysis reduces the dependence on conventional energy sources (such as fossil fuels), the volume of PET waste, and the carbon footprint of plastic products by reducing carbon monoxide and carbon dioxide emissions [26]. However, pyrolysis produces detrimental materials such as biphenyls, which create environmental pollution and health problems [27]. The substrates required for biological upcycling in this process are expensive as they require purification.

## Substrate Production by PET Gasification

Gasification is the degradation of plastics at high temperatures (700-1300°C) using oxidizing agents such as oxygen, steam, and air to produce syngas or producer gas consisting of methane, carbon monoxide, and hydrogen gas [28]. Depending on the gasifying agent and heterogeneous char gasification reactions, gasification involves drying, devolatilization, tar cracking, combustion, and shifting [28]. This process is mainly affected by factors including the type of plastic (aliphatic or aromatic), bed material, temperature, and gasifying agent [29]. Depending on the oxidizing agent, steam gasification, air gasification, and co-gasification, wherein different compounds are mixed before gasification, have been explored. Dolomite is used in the fluidized bed to reduce agglomeration and coke formation on active carbon. Active carbon is predominantly used as a tar remover, improving syngas quality [30]. Steam gasification uses steam as the primary hydrogen source. Considering the effect of the operating conditions on the tar and gas composition during the steam gasification of PET, increasing the temperature improves the hydrogen and carbon monoxide yields as the residence time and steam-to-fuel ratio are increased; and carbon monoxide and carbon dioxide contents are decreased [28]. Air gasification of PET was performed in a two-stage gasifier with active carbon. Air was used instead of steam to provide hydrogen and carbon monoxide, yielding producer gas (90.93 wt%), char (6.15%), tar (2.90%), and condensate liquid (0.03 wt%)[29]. Gasification is considerably flexible in treating various feedstock composites and can be integrated into current energy and fuel production systems. However, PET exhibits low effective carbon conversion, which reduces syngas heat. During steam gasification, less than 30% of carbon in PET is converted into gaseous products at 700°C. This conversion is required to form CO_2_, which is an important constituent of syngas; however, PET produces 3-4 times less carbon dioxide [28].

## Substrate Production via Plastic Depolymerization

depolymerization is the application of polymer chemistry to undo polymerization reactions to yield PET monomers, *e.g.*, bis(2-hydroxyethyl) terephthalate (BHET) and mono(2-hydroxyethyl) terephthalate (MHET) by glycolysis, a typical chemical depolymerization process. The product portfolio can be altered according to the depolymerization method used, which may include chemical, biological, or chemoenzymatic processes. Therefore, in this section, we review the recently developed status and product portfolios along with the advantages and disadvantages of individual depolymerization to select a suitable process for upcycling.

## Chemical Depolymerization

Chemical depolymerization uses chemicals and catalysts to influence the breakdown of PET into its monomers, BHET, MHET, TPA, and EG obtained from glycolysis, DMT from methanolysis, and TPA derivatives from aminolysis. Chemical depolymerization methods include alcoholysis (methanolysis), aminolysis, hydrolysis (steam, mineral acids, water, and alkalis), and glycolysis [31]. Inorganic catalysts, organocatalysts, and ionic liquids have been used to accelerate depolymerization.

Aminolysis involves the reaction of PET in primary amine-rich solutions, namely, hydrazine, ethanolamine, methylamine, allylamine, and ethylamine, to produce TPA and EG diamides. PET is used in the form of powder or fibers (temperature range = 20-100°C) [32]. Waste PET bottles were converted into hydrogel adsorbents through aminolysis. PET was reacted with tri- and tetraamines, diethylene amine, and diethylene-tetraamine, producing monomers, dimers, and oligomers, respectively, which were interlinked with ethylene glycol diglycidyl ether to form hydrogels [33]. Additionally, using an ultrafast microwave, aminolysis of PET could be conducted without catalysts in the primary amine-rich solutions at 180-200°C. Consequently, terepthalamides with different functional groups, namely, dihexylterephthalamide (DHTA), bis-(2-hydroexylethylterephtalamide (BHETA), bis-(furan-2-ylmethyl terepthalamide (BFTA), and diallyterepthalamide (DAA) were produced. These terepthalamides were used as plasticizers for polylactide and polylactic acid to reduce brittleness or as resin components for photopolymerizable film production [34]. PET was reacted with 1,2-diamino propane at temperatures in the range of 100-130°C for 20-24 h, yielding a combination of products, namely, monomers, dimers, trimers, and oligomers; the reaction of the monomers with salicylaldehyde produced a Schiff base, which can be used as a precursor for biologically active ligands, complexes, and catalysts [35]. Aminolysis requires minimal energy and time, and a simple purification step for the synthesized products. Because of these advantages, we expect that BHETA, DHTA, BFTA, and DAA could potentially be applied in biological upcycling for synthesizing high value-added chemicals after further depolymerization into TPA and EG, with simultaneous catabolism of certain microorganisms.

In methanolysis, PET is degraded by methanol at high temperatures (160-300°C) and pressures of up to 7 MPa in the presence of transesterification catalysts, consequently forming DMT and EG as the primary products. Additionally, PET oligomers (dimers, trimers, and tetramers) are possibly formed. Methanolysis involves two steps: depolymerization and purification of DMT through crystallization and distillation [36]. Methanolysis is influenced by factors including type and amount of catalyst, temperature, and time. PET waste methanolysis was done at 200°C for 2 h with bamboo leaf as a green heterogeneous catalyst; DMT and EG were the primary products with yields of 78% and 76%, respectively [37]. PET was also depolymerized with methanol in the presence of calcined sodium silicate at 180-200°C for 30 min, resulting in DMT (95% yield) and EG (depolymerization rate = 100% [38]. Using poly ionic liquids, namely, PIL-Zn^2+^ and PIL-Co^2+^ as catalysts, PET was depolymerized with methanol into DMT and EG under optimized conditions, resulting in DMT (89.1% yield) with 100% PET conversion [39]. PET was depolymerized at 200°C for 30 min using MgO/NaY (4 wt%), producing DMT (91%yield) and EG with 99% conversion of PET [40]. A low-energy catalytic methanolysis process was developed to depolymerize PET using methanol, with potassium carbonate as a catalyst, at 20-35°C. A high yield of 93.1% DMT and EG was obtained at 25°C, indicating that methanolysis could be performed at low temperatures [41]. Relatively low-quality PET can be used for methanolysis because of a simplified purification process involving the DMT product. Moreover, increased levels of contamination are tolerated, thereby offsetting chemical processing costs. However, methanolysis is expensive and sensitive to water presence, which is linked with catalyst poisoning [36]. DMT cannot be directly used in biological upcycling but can be converted into TPA, which is the required substrate. DMT was hydrolyzed to TPA in the presence of Nb/HZSM-5, a solid acid catalyst in the range of 160-220°C. The conversion was influenced by temperature changes; the DMT conversion and TPA yield increased gradually with an increase in temperature. At 160°C, DMT conversion and TPA yield were 33.4 and 21.8%, respectively; at 200°C, the DMT conversion was complete and the rate was 100% (DMT yield = 93.5%) [42]. Similar to the aminolysis products (BFTA, BHETA, DAA, and DHTA), the methanolysis product, DMT, can be applied to biological upcycling as a substrate after applying a suitable hydrolysis process to produce TPA and EG from DMT.

Glycolysis is a significant chemical depolymerization method and is used to obtain BHET and EG, which are used in bio-upcycling. Glycolysis is typically performed on a commercial scale and is consistently mediated by trans-esterification catalysts using EG as a solvent at a high temperature range (180-250°C), producing BHET, oligomers, and EG [43, 44]. Glycolysis uses different types of catalysts, namely metal-based ceramics, biomass-derived materials [45], organocatalysts [46], metallic chlorides, acetates, enzymes, and eutectic solvents based on ionic liquids and metal salts [47, 48]. Catalysts influence the speed of glycolysis; however, different catalysts have different costs and affect the process following glycolysis by influencing the enzymes/cells used, thereby requiring purification and increasing the economic cost of the entire process. Catalysts typically harm the environment; hence, biomass-derived materials are currently being investigated. Glycolysis is a simple, low-cost, and flexible method that produces a high yield of BHET monomer [47]. PET was depolymerized in the presence of EG and various catalysts, namely, graphite carbon nitride, melamine, potassium chloride, and sodium chloride at 160-96°C for 5-120 min; using graphite carbon nitride nanocatalysts resulted in BHET with 80.30% yield. Owing to better yields, lower total costs, and pollution reduction, graphite carbon nitride nanocatalysts are superior to metal-based and metal-free ionic catalysts [46]. Virgin PET pellets were depolymerized via glycolysis to estimate the efficiency of BHET production with modified conditions: temperature = 180-220°C and time = 10-180 min. PET was glycolyzed in the presence of a pure zinc acetate catalyst at a pressure of 3 bar, producing oligomers, dimers, and BHET at 180 and 220°C in short periods (e.g., 10 min) [49]. Because glycolysis depends on temperature and time, determining the optimum conditions is important. Post-consumer PET samples were broken down through glycolysis in the presence of deep eutectic solvents: choline chloride-thiourea and choline chloride-urea as catalysts, producing residual PET which was hydrolyzed in the presence of sodium carbonate and EG for a microwave irradiation time of 3 min; TPA, MHET, and BHET were obtained with yields of 62.79-80.66, 17.22-34.79, and 0.54-0.59%, respectively, with 99% PET conversion. Microwave irradiation was used because it reduces the reaction time, proving that PET can be rapidly depolymerized [50]. PET from a soft drink company was recycled using conventional heating and microwave radiation as energy sources in the presence of Lewis acids: t-BuNH_2_/LiBr in glycolysis and t-BuNH_2_/NaCl in hydrolysis. Conventional heating was performed at 197°C for 25 h, whereas microwave radiation was performed at 210°C for 30 min, producing EG and TPA. Microwave radiation is recommended as an energy-conserving process because PET is completely depolymerized within a short duration [51]. An oyster-derived (biomass-derived) catalyst was used in glycolysis optimization, which could break down PET at 195°C for 1 h into BHET and EG with 68.6% yield. By replacing conventional metal-based catalysts with biomass-based catalysts, an environmentally friendly and economically competitive glycolysis process can be established [45]. Glycolysis has several advantages, including simplicity, flexibility, low cost, and the synthesis of TPA, EG, and BHET, which can be used in the upcycling process with or without purification [52]. However, this process involves high pressure and temperature, catalysts, and difficulty in separating and purifying oligomers from the desired products [53].

## Biological Depolymerization

Biological depolymerization produces intermediates by biologically degrading plastic, which is subsequently subjected to downstream bioconversion to obtain value-added products. This process generally uses a PETase-MHETase dual enzyme or a single enzyme system. Enzymatic depolymerization involves the use of enzymes with promiscuous activities, such as lipases, esterases, cutinases, carboxyl esterases, and MHETase, to release BHET and MHET monomers, which are further degraded to TPA and EG as the final products [47, 54]. These enzymes have been discovered in various microbes, such as *Bacillus* sp. [55, 56], *Fusarium* sp. [57, 58], *Thermobifida* sp. [59, 60], *Pseudomonas* sp. [56], and *Saccharomonospora* sp. [61], which have been used to degrade PET into its monomers.

*Is*PETase (from *Ideonella sakaiensis*) and leaf branch compost cutinase (LCC) are the most promising enzymes discovered thus far. Cutinase was found originally in plant-infecting fungi that degraded cutin, an insoluble polymeric plant structural compound, for an effective invasion [62, 63]. LCC belongs to the α/β hydrolase family, with a classical serine-histidine-aspartate catalytic triad often found in lipases and esterases [64]. The aspartate alters the pKa of the histidine to act as an effective acid/base, which in turn facilitates deprotonation of the serine. The deprotonated serine can exert nucleophilic attack on the ester bond of the substrate, forming a tetrahedral acyl intermediate. A water molecule activated by the histidine can then hydrolyze the acyl-enzyme bond. PETase is a serine hydrolase that shares a similar reaction with cutinases and attacks PET polymer releasing TPA, BHET, and MHET [65]. MHETase hydrolyzes soluble MHET, generating TPA and EG. According to many crystal structure and biochemical studies, *Is*PETase has an open active site that can bind to PET oligomers [1, 66]. PETases are generally mesophilic with the optimum temperature at 30-37°C, whereas LCC is thermostable, with the maximum productivity at 72°C, and has the highest PET-to-TPA degradation productivity reported thus far (up to 16.7 g/L/h) [67]. Hydrolases with amorphous PET-degrading abilities at high temperatures approaching the glass transition temperature have also been discovered in thermophilic *Actinomycetes* [68], fungi [62], and plant compost [69]. These hydrolases were further engineered to increase their catalytic activities by introducing rationally designed site-specific mutations, by addition of surfactants that bring the enzyme to the PET surface, or by narrowing the binding site of the PETase by introducing cutinase-conserved residues [70-72]. The key strategies to enhance the activity involve either increasing the accessibility of the crystalline substrate to the active site, or adapting and optimizing the active site configuration to the specific substrates.

Several microorganisms with PET-degrading abilities, such as *Pseudomonas*, *Escherichia*, and *Bacillus*, have been discovered, engineered, and used for degrading PET into high-value chemicals such as vanillin, catechol, adipic acid, and muconic acid (MA) [73]. Among the *Pseudomonas* genus, *Pseudomonas putida* is generally used because it metabolizes PET polymer varieties as the sole carbon source owing to its high tolerance and metabolic ability. PET monomers such as EG and TPA are metabolized [69]. *Pseudomonas umsongensis* GO16 can use PET as the sole carbon source [74]. Other microorganisms have been engineered to produce enzymes with PETase activity from *Yarrowia lipolytica*, a yeast capable of hydrolyzing BHET from PET into TPA and EG [75]. *Bacillus megaterium* forms a biofilm on the PET film, which subsequently hosts *Rhizobium* sp., degrading PET into BHET [1]. *P. umsongensis* GO16, capable of degrading PET waste, was used to break down PET into TPA, which was further converted to hydroxy alkanoyl oxy-alkanoate (HAA) and EG, which can be synthesized into a polyhydroxyalkanoate polymer (PHA) [73]. Post-consumer PET waste was depolymerized by *Humicola insolens* to degrade PET without pre-treatment in moist solid reaction mixtures with 3 wt% of the enzyme, and sodium carbonate as a catalyst, producing TPA with 49 ± 2% yield [76]. PET was depolymerized using PETase-RolA at 30°C into BHET and MHET with a PET weight loss of 26% over 4 days. The PET monomers were further broken down into TPA and EG. Hydrolysis of PETase was increased by hydrophobic RolA [77]. *Is*PETase variants were used to depolymerize PET films at 30 and 40°C, producing MHET and TPA. Among all PET-degrading enzymes, *Is*PETase exhibits high stability and the highest PET degradation activity under mild conditions; however, it has low thermal stability [78]. Highly efficient and optimized PET hydrolase from *Thermobifida fusca* was used to depolymerize amorphous PET for over 10 h yielding 16.7 g/L/h TPA with >90% PET depolymerization [79]. An artificial microbial consortium composed of *Rhodococcus jostii*, *P. putida*, and two metabolically engineered *Bacillus subtilis* capable of producing PETase and MHETase was used to degrade PET films, causing a 23.2%weight loss within 7 days and yielding TPA and EG. Using a microbial consortium can reduce the metabolic burden, minimize TPA and EG inhibition effects, and promote biodegradation. Combining more microorganisms improves the degradation rate and efficiency [80]. TPA is degraded by *Rhodococcus* sp. SSMI is the sole carbon both in the presence and absence of the PET film, thus increasing the amount of TPA in the presence of PET films, which indicates that the films have been degraded into TPA [81].

Therefore, biological depolymerization is an environmentally friendly method because it uses mild conditions (temperature and pH) in the absence of hazardous chemicals [47]. Enzymes are specific and selective to specific substrates, rendering the process effective, as they can be used to act on targeted substrates. Enzymatic depolymerization (e.g., hydrolysis) is crucial for producing substrates suitable for bioconversion (e.g., TPA and EG). However, biological depolymerization incurs high operating costs [82]. To establish economically feasible biological depolymerization processes, possible further developments include reducing the enzyme production costs and improving the catalytic properties and stability.

## Chemoenzymatic Depolymerization

Several combined chemoenzymatic depolymerization processes have been developed, wherein chemical depolymerization, including glycolysis, yields BHET, oligomers, and EG, and enzymatic hydrolysis breaks down BHET and MHET into TPA. TPA and EG are the substrates used for bioconversion. PET was glycolyzed in the presence of urea/NaOAc·3H_2_O, a eutectic solvent-based catalyst, and EG to obtain the main product: BHET (35%yield and 73.6% PET conversion). The synthesized BHET was further hydrolyzed by *Candida antarctica* lipase B to obtain TPA with an overall yield of 57% [47]. PET granules were broken down in a single pot in the presence of a biocompatible catalyst, betaine, and EG, yielding BHET and EG, which were subsequently converted into TPA and EG. These products were converted by the whole cells of *Escherichia coli* and *Gluconobacter oxydans* into protocatechuic acid (PCA) and glycolic acid (GLA), respectively. Betaine facilitated chemo-glycolysis and enzymatic hydrolysis in one pot without purification, thereby reducing the economic cost [3]. Disposable PET bottles were depolymerized through glycolysis in the presence of a biocatalyst, potassium carbonate, and further hydrolyzed into TPA and EG using *B. subtilis* esterase. However, long reaction times resulting from the slow hydrolysis of MHET are caused by the MHET structure, which affects the enzyme-substrate complex; hence, enzyme structure-based protein engineering is recommended [8]. PET was glycolyzed in the presence of titanium (IV) butoxide, yielding BHET and EG, using *P. putida* AW 165; BHET was converted into 15.1 g/L of β-ketoadipic acid with 76% molar yield [83]. The chemoenzymatic depolymerization process utilizes the advantages of chemical glycolysis (flexibility, simplicity, and high yield of BHET) and enzymatic hydrolysis (selectivity and mild conditions) to produce TPA, which is a precursor for synthesizing high value-added chemicals.

## Production of High-Value Products from PET Monomers by Upcycling Processes

**Fig. 3:**
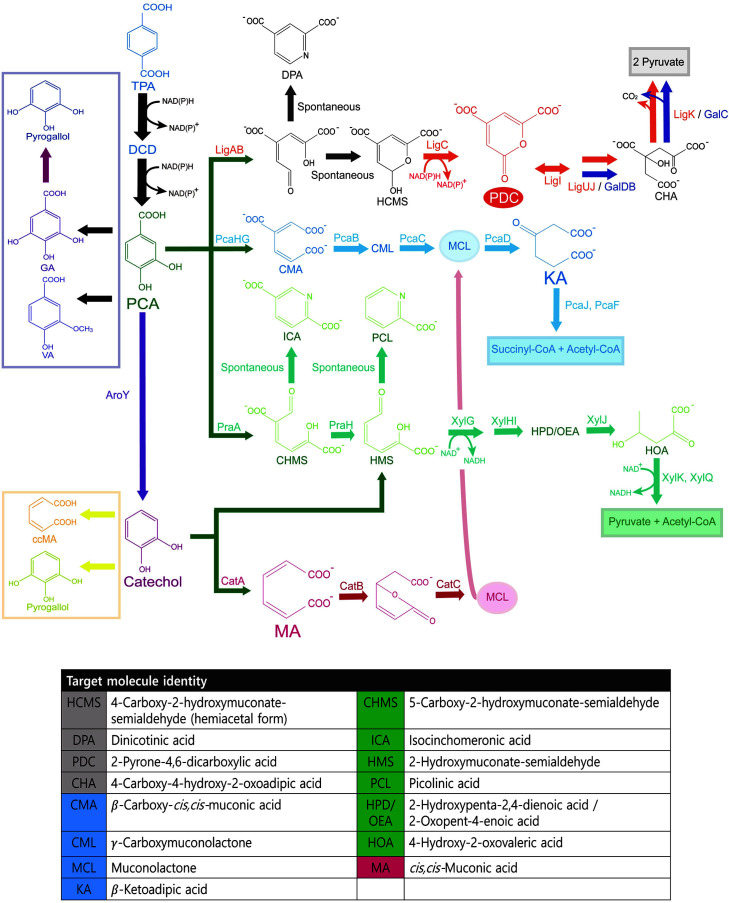
Catabolic pathways of TPA to aromatic chemicals: catechol, PCA, and other high value-added chemicals. The α-ketoadipate pathway (blue line) consists of genes, namely, PcaHG (protocatechuate 3,4-dioxygenase), PcaB (3-carboxy-cis, cis-muconate cycloisomerase), PcaC (γ-carboxy-muconolactone decarboxylase), and PcaD (β-ketoadipate enol-lactone). The targeted products include: CMA, β-carboxy-cis,cis-muconic acid, CML-γ-carboxymuconolactone, MCL, Muconolactone,KA,β-ketoadipic acid. The PCA 2,3-cleavage pathway (light green lines) consists of genes, namely, PraA, (PCA 2,3-dioxygenase), PraH, (5 CHMS decarboxylase), 5C-2HMS dehydrogenase, XyHI,4-oxalocrotonate isomerase, XyIJ,2- oxopent-4-enoate hydratase,XyIK, 4-hydroxy-2-oxovalerate aldolase, XyIQ, acetaaldehyde dehydrogenase. The targeted molecules are: CHMS, 5-Carboxy-2-hydroxymuconate-semialdehyde, ICA, isocinchomeronic acid, HMS, 2-Hydroxymuconate semialdehyde, PCL-picolinic acid, HPD/OEA, 2-Hydroxypenta-2,4-dienoic acid/2-oxopent-4-enoic acid, HOA, 4-Hydroxy- 2-oxovaleric acid. The other pathway (grey line) is encoded by LigAB,4,5-PDC,Lig C,CHMS dehydrogenase, Lig I, PDC hydrolase, LigJ, OMA hydratase, LigK, CHA(4-carboxy-4-hydroxy-2-oxoadipate) aldolase yields energy compounds. The targeted compounds include: HCMS, 4-Carboxy-2-hydroxymuconate-semialdehyde, DPA-dinicotinic acid, PDC, 2-pyrone- 4,6-dicarboxylic acid, CHA, 4-Carboxy-4-hydroxy-2-oxoadipic acid. Catechol is catabolized by CatA, Catechol 1,2- dioxygenase,CatB, Muconate cycloisomerase and Cat C, Muconolactone isomerase into MA, cis,cis-Muconic acid.

Upcycling involves the conversion of degraded products from chemical transformations or biological degradation into various value-added biochemicals via chemical or biological routes [84]. Substrate production processes for upcycling using various degradation methods, such as pyrolysis and gasification, have been efficiently developed. However, because recently published research is predominantly related to upcycling BHET, TPA, and EG, we focus on the upcycling status of these chemicals. Therefore, in this section, after reviewing the biological upcycling of TPA and EG by TPA/EG-metabolizing microorganisms and whole-cell conversion into high-value-added chemicals, as shown in Fig. 3 and Table 2, we suggest a promising convergence process for upcycling PET.

## Biological Upcycling of TPA or EG by TPA- or PET-Metabolizing Microorganisms

TPA, a PET hydrolysis product, is not widely regarded as a bacterial growth substrate, due to its toxicity to cells [74]. However, PET biodegradation using *I. sakaiensis*, which could simultaneously depolymerize PET and catabolize the hydrolysis products:TPA and EG, was first reported in 2016 [85]. Thus, PET is no longer regarded as a non-degradable plastic in the ecosystem. Although TPA catabolism has been previously reported in various environmental microorganisms, such as *Comamonas*, *P. putida*, and *Thermobifida* sp., the discovery of *I. sakaiensis* has accelerated the development of biological upcycling. Typical TPA catabolism progresses to the common metabolite, PCA, wherein TPA is converted into PCA after being encoded by *tph* genes in two catabolic steps: i) addition of two hydroxyl groups at positions 4 and 5 of TPA by the activity of TPA dioxygenase (TphA12A3), producing 1,6-dihydroxycyclohexa-2,4-diene dicarboxylate (DCD), and ii) removal of the carboxyl group at position 6 by the action of 1,2-dihydroxy-3,5-cyclohexadiene-1,4-dicarboxylate dehydrogenase (TphB). The *tph* gene cluster was found in *Rhodococcus* sp. strain DK17, *Comamonas* sp. strain E6, and *Comamonas* testosteroni YZW-D. These genes encode a tphR transcriptional regulator, an ICIR-type activator that responds to the inducer TPA. Oxygen-dependent TPA dioxygenase and NAD(P)H, flavin, and iron-sulfur-dependent reductase catalyzes the first dihydroxylation. The final reductive decarboxylation of DCD by a zinc-dependent dehydrogenase yields PCA [86,87]. PCA was further catabolized to various common metabolites, such as acetyl-COA, succinyl-COA, pyruvate, and oxaloacetate, which are used for energy metabolism (Fig. 3) [88].

**Fig. 4:**
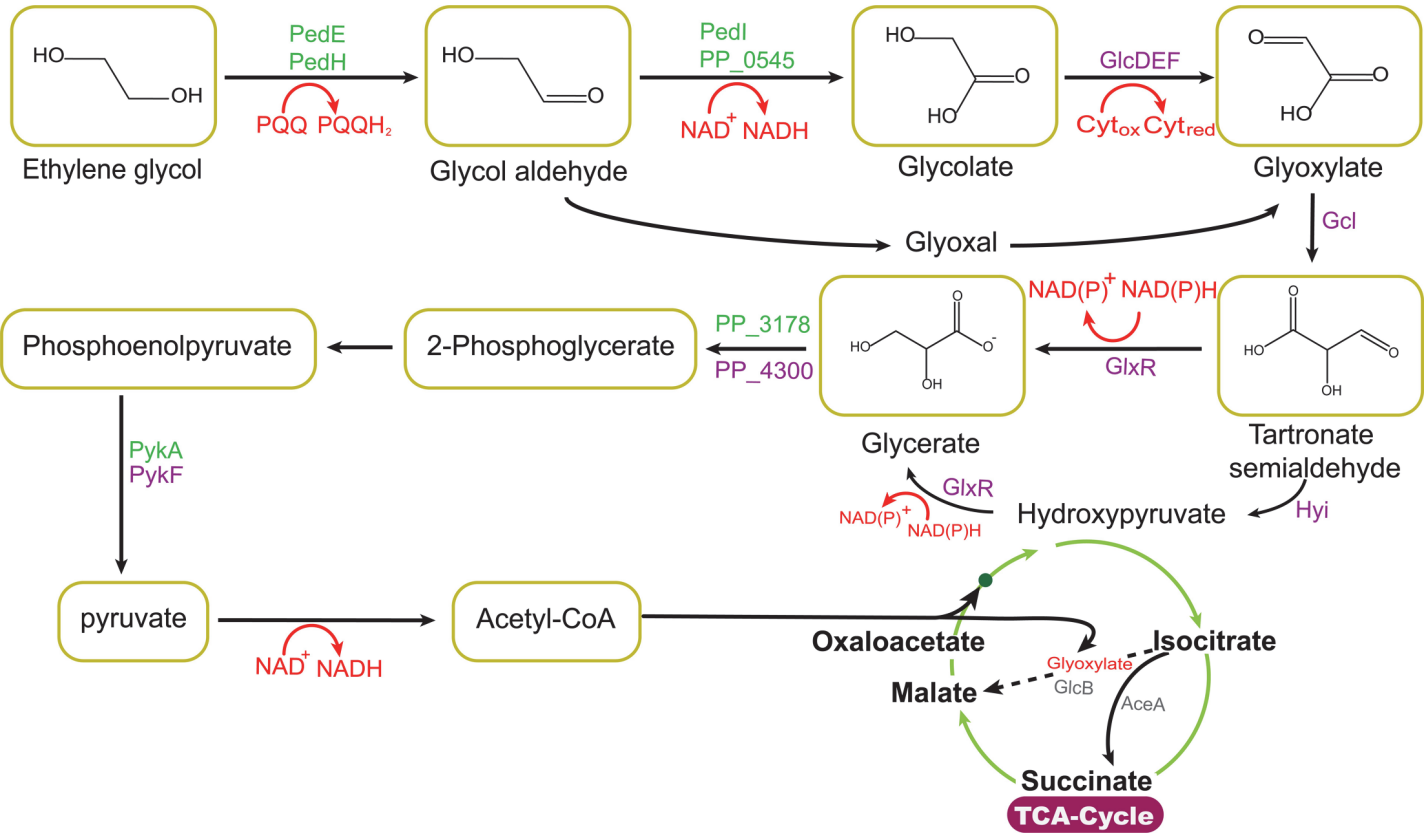
Metabolic pathway of ethylene glycol to glyoxylate and other energy compounds. This consists of PQQ (pyrroloquinoline quinone), which is reduced to PQQH_2_ upon conversion of ethylene glycol to glycolaldehyde and other compounds synthesized from glycolate. The enzymes shown are PedE/PedH, PQQ-dependent alcolhol dehydrogenase, Pedl, peroxisomal 3-ketoacyl-COA thiolase, GlcDEF, glycolate dehydrogenase, GlxR, tatronate semialdehyde reductase, Hyi,hydroxypyruvate isomerase and PykA/Pyk, pyruvate kinase.

EG is metabolized via various pathways, namely oxidization to ethanol and acetaldehyde, which are converted to acetate via acetyl-CoA, followed by substrate-level phosphorylation to form ATP (Fig. 4). Ethanol can be oxidized and the reducing equivalents can be reused by CO_2_ reduction to acetate in the Wood-Ljungdahl pathway. EG can be catabolized to acetaldehyde by propanediol dehydratase (PduCDE) and CoA-dependent propionaldehyde dehydrogenase (PduP) proteins encoded by the *pdu* gene and by CoA-dependent oxidation to acetyl-CoA [89]. EG is broken down via the synthesis of glyoxylate, which is further catabolized into pyruvate, succinate, and oxalate [90]. EG metabolic activities have been found in various microbes, including *E. coli* with propanediol oxidoreductase for metabolizing short-chain alcohols and aldehydes, *P. putida*, and *Pseudomonas aeruginosa* with periplasmic alcohol dehydrogenases [86]. EG was synthesized into medium-chain polyhydroxyalkanoate (mcl-PHA) using the engineered *P. putida* MFL 165 strain. This strain was engineered with various genes, namely glyoxylate carboligase (*gcl*), hydroxy pyruvate isomerase (*hyi*), pyruvate kinase (*pykF*), tartronate semi-aldehyde reductase (*glxR*), and hydroxypyruavte reductase (*ttuD*), which enabled the strain to grow on EG, converting it to mcl-PHA with a yield of 0.06 ± 0.00 g [69]. EG metabolism provides high-value chemicals such as PHA and GLA; however, EG is also toxic to the enzymes and cells used in its metabolism [90]. PET waste was hydrolyzed into TPA and EG hydrolysates, which were subsequently metabolized by *P. umsongensis* G016 KS03 to yield a fatty acid derivative HAA. The cell is equipped with an rhlA gene, acyltransferase, which instigates the two activated fatty acids’ esterification to HAA [91].

Although TPA or EG can be easily converted into target chemicals by metabolic engineering, several drawbacks include a low growth rate [85] and limited engineering tools [92] for direct application to microbial chassis for producing value-added chemicals. Future studies can focus on chassis engineering to improve growth and productivity, remove unnecessary metabolic pathways, and reinforce substrate utilization capability to generate industrial strains.

## Whole-Cell Conversion of TPA and EG

The whole-cell conversion uses engineered cells containing TPA-metabolizing genes or wild-type cells with the ability to degrade TPA, thus enabling the conversion of TPA and EG into other chemicals for further use. Whole-cell microbial catalysts containing *E. coli* expressing metabolizing enzymes were used to convert TPA into various value-added chemicals like MA, gallic acid (GA), PCA, catechol, pyrogallol, and vanillic acid (VA) through the TPA degradation pathway, with yields of 32.7-92.5%. The whole-cell substrate loading and carbon yield were satisfactory, thus affecting the overall yield of the chemicals produced. The resulting chemicals can be utilized for manufacturing sanitizers, pharmaceutical products, cosmetics, animal feeds, and bioplastic monomers. The TPA metabolizing pathway involves a combination of hydroxylation, methylation, decarboxylation, and oxidative ring cleavage with two enzymes: TPA 1,2-dioxygenase converting TPA to DCD and DCD dehydrogenase converting DCD to PCA. The TPA transporter in whole cells has not been extensively studied; additionally, it affects the total substrate loading. PCA was the first precursor, which was subsequently converted into other aromatic-derived chemicals: VA, MA, GA, and pyrogallol. EG was converted by *G. oxydans* KCCM 40109 into glycolic acid, which can be used as a cosmetic ingredient [13]. Engineered *E. coli* strain PCA-1 expressed with TphAabc and TphB encoding TPA 1,2-dioxygenase and DCD dehydrogenase produced 3.8 g/L of PCA from 4.5 g/L of TPA with a 90.4% molar yield; using *G. oxydans*, 31.4 g/L of GLA was obtained from 30.6 g/L EG with a molar yield of 91.6%(mol/mol) [3]. Engineered *E. coli* and a thermostable enzyme LCC variant (WCCG) were used as biocatalysts for upcycling PET-derived monomer TPA directly into vanillin, a high value-added compound, which is commonly used in the cosmetic and food sectors as a bulk chemical. With process optimization, 79% of vanillin was converted from TPA. Although 68 μM of vanillin was detected, the absence of LCC resulted in lower levels of vanillin possibly because of background PET hydrolysis in the LCC reaction [67]. TPA was obtained by microwave-aided hydrolysis using a biomass-acquired SiO_2_ catalyst with thiol functionalization and further hydrolyzed to 2-pyrone-4,6-dicarboxylic acid with recombinant *E. coli* strains [93].

Whole-cell conversion is cost-effective and less time-consuming, with no enzyme purification; rapid validation of new synthetic pathways is possible. However, this process exhibits a shortcoming of low substrate loading; hence, chassis engineering, adaptive laboratory evolution, and the introduction of heterologous TPA transport systems are required [94]. Further studies considering substrate loading improvement by transporter introduction and chassis engineering enabling the endurance of high TPA loading are recommended.

## Development of Convergence Technology for PET Waste Upcycling

**Fig. 5:**
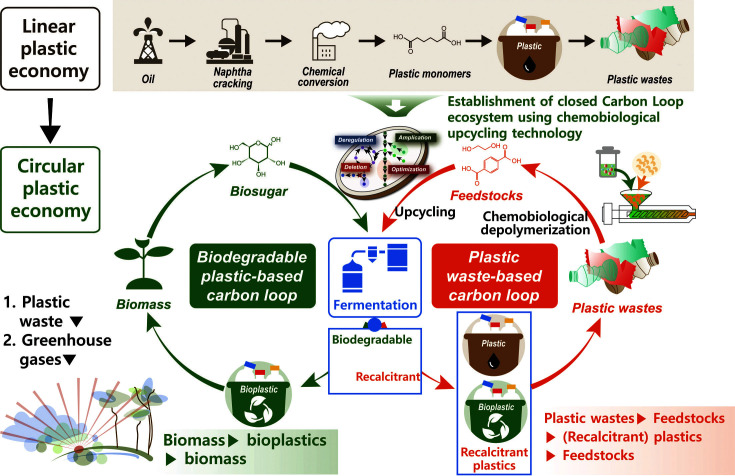
Lifecycle of plastics, including the linear economy, wherein plastics from fossil fuels end up as plastic waste in the environment, and the circular economy, wherein plastic waste from fossil fuels undergoes various processes to yield high value-added chemicals, thus conserving the environment.

Plastic upcycling technologies are instrumental in solving current plastic waste problems, in addition to biodegradable plastic production technology, because all conventional plastics cannot be replaced by biodegradable plastics (Fig. 5). Although disposable plastics for packaging should be replaced with biodegradable plastics, the long-term use of plastics with less biodegradability, such as plastics for processed food and electronics, would continuously generate potential plastic waste. The development of plastic upcycling technologies consists of two important steps: i) substrate production for bioconversion via PET waste depolymerization, and ii) upcycling of the produced substrates.

The depolymerization of PET is essential because it provides substrates that can be used in biological upcycling to produce high value-added chemicals through a circular loop of plastic upcycling, thereby reducing the amount of plastic in the environment. Chemical depolymerization breaks down PET waste into BHET, which can be used as a substrate in enzymatic depolymerization. This method has an increased tolerance for high levels of contamination, and lower-quality PET can be used. However, high reaction conditions (high temperatures and pressures) and expensive catalysts increase the overall cost of the process and produce low-purity target chemicals, for example BHET, including lots of unnecessary chemicals due to additives and side reactions. Besides, the produced substrates require transformation to be applied for bioconversion. The produced substrates require transformation to be applied for bioconversion. Biological depolymerization can be developed as an alternative to chemical depolymerization with various advantages: operation at low temperatures, no pressure, and selective depolymerization of PET waste from mixed plastic waste. However, significant disadvantages are observed in terms of operating costs, including enzyme production. Recently, chemoenzymatic depolymerization has been considered an efficient method to produce substrates for the bioconversion of PET waste, because it adopts the advantages of chemical and biological depolymerization processes. Through chemical depolymerization, a substantial amount of PET waste can be easily transformed into smaller chemicals such as BHET. The products can then be transformed into suitable substrates for bioconversion by enzymatic hydrolysis processes using enzymes such as esterases. In the future, the development of more efficient chemical depolymerization methods using biocompatible catalysts with advanced processes, along with a singular enzymatic system operating at a biocompatible temperature (e.g., 37°C) and improved hydrolysis capability, can provide the economic feasibility to contribute to plastic waste reduction.

Several chemical and biological upcycling processes have been reported thus far. Considering BHET, chemical upcycling is advantageous because it can efficiently produce valuable items without further hydrolysis of BHET. For example, fiberglass-reinforced plastics were successfully developed by polymerizing BHET produced from PET waste with renewably sourced monomers, including acrylic acid, methacrylic acid, and MA [95]. However, the product portfolio to produce value-added chemicals is limited compared with that of the biological upcycling process; biological upcycling provides better benefits because it can directly produce various value-added chemicals by combining multiple enzymes. Biological upcycling involves the application of PET monomers, TPA, and EG, using whole-cell conversion and TPA-metabolizing microorganisms, to convert them into expensive chemicals. Depolymerization products are converted into high value-added products via biological upcycling. Moreover, it enables the circular utilization of PET, reduces the amount of plastic waste produced, and encourages the proposed circular economy to ultimately solve the prevalent greenhouse gas emission and crude oil consumption problems. However, biological upcycling is affected by limitations such as low substrate loading, and fewer known PET-degrading microorganisms. Therefore, additional studies on TPA/EG-metabolizing microorganisms, consolidated bioprocessing (CBP) for reducing enzyme usage, and advanced heterologous TPA transport systems toward efficient TPA uptake will be required.

Although the use of petroleum-based TPA and EG is more economically feasible, the continuous use of petroleum fossil fuels causes serious damage to the environment. Meanwhile, due to the reduction in fossil fuel reserves [96], TPA/EG synthesis has shifted from fossil fuels to biomass [97], hence creating further possibilities, including the suggested use of TPA/EG from recycled PET. Therefore, the application of TPA and EG from recycled PET enables the use of PET in a circular economy producing high value-added chemicals and reducing the plastic burden. The TPA/EG amounts currently obtained are so low that further research is needed to find more reliable microorganisms to break down PET into TPA/EG while also increasing the produced amounts to encourage a shift from laboratory scale to industrial scale.

## Conclusions

In this review, the current status of PET upcycling approaches was comprehensively addressed. Different forms of substrate fabrication using chemical, biological, and chemobiological methods were described. Subsequently, the value-added chemical production process by biological upcycling was discussed. However, considering the economic feasibility, each process presents disadvantages in terms of establishing a circular plastic economy. In the future, advancing individual technologies, including the production of substrates for bioconversion and biological upcycling, should be considered along with integrating each technology to design an economically feasible PET upcycling process. Based on this development, further studies should be conducted to develop and take practical technologies from the laboratory scale to an industrial scale to reduce PET waste and provide economic profit.

## Figures and Tables

**Table 1:** Degradation product types and yields obtained from PET using various PET degradation processes.

Process	Substrate	Product	Yield	Reference
Pyrolysis	PET bottle sheets^1^	Solid Gas	35.67 wt% 40 wt%	[22]
	PET water bottles with rice husk	Gas Biocrude char	39.9-68.9 wt% 12-29% 15.7-31.7%	[23]
	PET bottles^2^	Gas Oil Solid residue	13.3±0.6 wt% 46.7±1.9 wt% 39.7±1.6 wt%	[24]
	Pure PET pellets	Gas: CO, CO_2_ Solid: TPA	- 25%	[25]
Gasification	Virgin PET pellets	Gas: H_2_, CO_2_, CO Char Tar	90.93 wt% 6.15 wt% 2.9 wt%	[29]
	Virgin PET pellets	Gas: H_2_ CO_2_ Biphenyl	-	[28]
**Depolymerization**				
Aminolysis	Post-consumer	PET DHTA BHTA BFTA DAA	64% 91% 82% 61%	[34]
	PET flakes	Monomers, Dimers, Trimers, Oligomers	-	[35]
Methanolysis	PET	DMT	89.1%	[39]
	PET	DMT EG	78% 76%	[37]
	PET	DMT EG	95%	[38]
	PET	DMT EG	91%	[40]
	PET	DMT EG	93.5%	[41]
Glycolysis	PET	BHET	80.30%	[46]
	PET	BHET	70%	[49]
	Post-consumer PET bottles	TPA MHET BHET	62.79-80.66% 17.22-34.79% 0.54-0.59%	[50]
	PET bottles	BHET, TPA, EG	Quantitative	[51]
Biological depolymerization	PET	BHET EG	68.6%	[45]
	Post-consumer PET waste	TPA	49±2%	[76]
	Amorphous PET	TPA	16.7 g/L/h	[79]
Chemoenzymatic depolymerization	PET	BHET MHET PET oligomers	84.8% 7.7% 8.7%	[8]
	PET	BHET	35%	[47]
	PET	TPA EG	99.9%	[3]

Note: DHTA, dihexylterephthalamide; BHTA, bis(2-hydroxyethyl) terepthalamide; BFTA, bis(furan-2-ylmethyl) terepthalamide; DAA, diallyterepthalamide; DMT, dimethyl terephthalate; TPA, terephthalic acid; BHET, bis(2-hydroxyethyl) terephthalate; MHET, mono-(2-hydroxyethyl) terephthalate; EG, ethylene glycol.

1-solid products contained benzoic acid, 4-vinyl benzoic acid, monovinyl terephthalate, and divinyl terephthalate.

2-Oil components include paraldehyde (54.7 wt%), ethylene glycol (23.65 wt%), and benzoic acid and benzoates (11.5 wt%). The solid carbonaceous residue contained carbon, ash, nitrogen, and sulfur). Gas contained CO and CO_2_ (more than 90 vol%), a few C1 -C 4 hydrocarbons (~7 vol%), and hydrogen (~3 vol%).

**Table 2:** Chemicals produced by whole-cell conversion.

Substrate	Recombinant organism	Products	Production yields	References
TPA	*E. coli*	PCA	81.4%	[13]
		Gallic acid	15.9%	
		Catechol	97.8%	
		Pyrogallol	39.0%	
		Vanillic acid	41.6%	
		Muconic acid	85.4%	
EG	*G. oxydans* KCCM 40109	Glyoxylic acid	98.6%	
TPA	*E. coli* PCA -1	PCA	90.4%	[3]
EG	*G. oxydans* KCCM 40109	Glyoxylic acid	91.6%	
TA	*E. coli* RARE-pVanX	Vanillin	79%	[67]
TPA	*P. stutzeri* TPA-3P	PHB	11.56 wt%	[75]
TPA	*P. umsongensis* GO16 KS3	HAA	35 mg/L	[73]
TPA	*P. putida* GO16, G019,	PHA, 3-hydroxydecanoic acid	4.4 mg/L/h	[74]
	*P. frederiksbergensis* (GO23)		8.4 mg/L/h	
TPA	*I. sakaiensis*	PHA	0.75 ± 0.09 g/L	[98]

Note: PCA, protocatechuic acid; PHB, polyhydroxy butyrate; HAA, hydroxy alkanoyl oxy-alkanoate; PHA, polyhydroxyalkanoate
